# Supplementary material for: Poison or Potion: Effects of Sunflower Phenolamides on Bumble Bees and Their Gut Parasite
Source: Biology (Basel). 2022 Apr 1;11(4):545. doi: 10.3390/biology11040545 (PMC9030180; doi:10.3390/biology11040545)

Figure S1. Ordination of the phenolamide profiling in sunflower tissues. The first two axes of the principal component analysis (PCA) are shown and express 89.2% of the total variance. Each point represents a replicate of plant tissues (one symbol per tissue) with superimposition of leaves and corolla (*i.e.*, no detected phenolamides). Each vector represents a variable (phenolamide compound) with N,N'-diferuloyl spermidine (1), N,N',N''-tricoumaroyl spermidine (2), N,N',N'',N'''-tetracoumaroyl spermine (3), N,N',N''-dicoumaroyl feruloyl spermidine (4) and N,N',N'',N'''-tricoumaroyl feruloyl spermine (5).

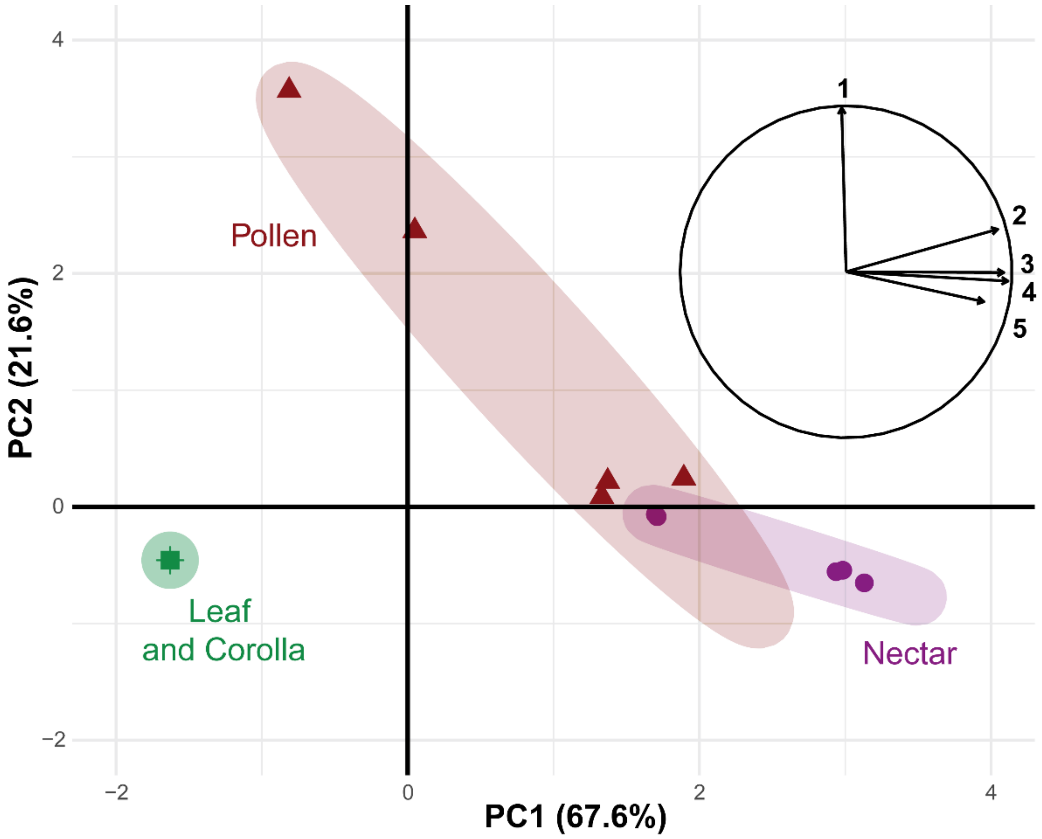

Supplement: Supplementary file 1 [file biology-11-00545-s001.zip › Figure S1.pdf]
