# Supplementary material for: Poison or Potion: Effects of Sunflower Phenolamides on Bumble Bees and Their Gut Parasite
Source: Biology (Basel). 2022 Apr 1;11(4):545. doi: 10.3390/biology11040545 (PMC9030180; doi:10.3390/biology11040545)
Supplement: Supplementary file 1 [file biology-11-00545-s001.zip › Protocol S1.pdf]

Protocol S1. Phenolamide analyses.

While pollen samples had already a powder aspect, dried sunflower leaves and petals were hammer grinded (6,000 rpm; Polymix® PX-MFC 90 D) prior to the extraction process. Samples (*ca.* 50 mg) were then suspended in 1 mL of a methanol/water (70:30 v/v) extraction solvent and vigorously bead beaten at 30 Hz for 2 min to disrupt the cell structure and to extract the phenolamides (five glass beads of 2 mm; Retsch® Mixer Mill MM 400). Following centrifugation at 4,500 rpm for 10 min (Sigma 2-16P), the supernatants were filtered using a 0.2 µm syringe filter (Pall Acrodisc Syringe Filter with Nylon Membrane, 13 mm) and 500 µL of the resulting solutions were accurately collected, dried at 55°C for three hours and weighed. Then the dried extracts were dissolved in 1 mL of methanol/water (70:30 v/v) solvent. Regarding nectar, samples were centrifuged at 1,000 rpm for 1 min (Sigma 2-16P) and 0.1 – 0.8 mg of the supernatants were then suspended in 200 µL of a methanol/water (70:30 v/v) extraction solvent. This solution was directly ready for injection. Phenolamide profiles of the different samples were characterised using LC-MS and LC-MS/MS on two different mass spectrometers. Phenolamides from pollen and vegetative parts were separated via a Phenomenex® Kinetex C18 EVO column (150 × 2.1 mm i.d., 100 Å particle size) using a Waters™ Alliance 2695 system and then analysed via a Waters™ Q-ToF US mass spectrometer, while phenolamides from nectar were analysed using a Waters™ Acquity UPLC H-Class system (HPLC mode) and a Waters™ Synapt G2-Si mass spectrometer for greater sensitivity. A binary gradient was performed at a flow rate of 0.25 mL min^{-1}. The mobile phase consisted of methanol (solvent A, Chem-Lab HPLC, gradient grade) and water (Milli-Q filtered) + 0.01% formic acid (solvent B, Chem-Lab, p.a. grade). The gradient program was as follows: A = 10%, B = 90% at t = 0 min; A = 30%, B = 70% at t = 6 min; A = 35%, B = 65% at t = 11 min; A = 50%, B = 50% at t = 18 min; A = 90%, B = 10% at t = 23 min; A = 100%, B = 0% at t = 25 min; A = 100%, B = 0% at t = 27 min; A = 10%, B = 90% at t = 30 min. The temperature of the column was maintained at 40°C and the autosampler at 20°C. Injection volume was 5 µL. Mass spectrometers operated in electrospray (ESI) negative mode over a mass range of 50 - 2,000 Da. Typical MS conditions were: capillary voltage -3.1 kV/-2.5 kV (Q-ToF US/Synapt), cone voltage -30 V/-40 V (Q-ToF US/Synapt), source temperature 120°C, desolvation gas temperature and flow 300°C and 500 L/h, respectively, and scan time 0.5 sec. Phenolamides were identified by studying collision-induced dissociation (CID) spectra in positive mode and comparing the data obtained with literature. Quantifications were performed using triferuloyl spermidine as internal standard (concentrations expressed as triferuloyl spermidine mg equivalent / sample g) in triplicates to account for analytical variability (assuming the same response factor between the extracted phenolamides and the triferuloyl spermidine) [1,2]. The standard of *N,N′,N′′*-triferuloyl spermidine was synthesised in the laboratory by mixing 40 mL of dicholoromethane (Chem-Lab, p.a. grade) containing ferulic acid (62.5 mM, Sigma-Aldrich) and spermidine (18.4 mM, Alfa Aesar) with 10 mL of a solution of *N,N′*-dicyclohexylcarbodiimide (DCC) (250 mM, Alfa Aesar) in dichloromethane. The mixture was stirred at room temperature for 24 hours in the dark. The solution was then filtered and the solvent was evaporated under reduced pressure. The product was purified by flash chromatography (Biotage SP). The column used was a Grace Reveleris C18 (12 g) model, the flow 15 mL/min and the solvents were methanol (solvent A, Chem-Lab HPLC, gradient grade) and water (Milli-Q filtered) + 0.01% formic acid (solvent B, Chem-Lab, p.a. grade). The solvent gradient was as follows: A = 10%, B = 90% at t = 0 min; A = 50%, B = 50% at t = 5 min; A = 90 %, B = 10% at t = 18 min; A = 100%, B = 0% at t = 24 min; A = 100%, B = 0% at t = 25 min; A = 10%, B = 90% at t = 28 min; A = 10%, B = 90% at t = 30 min.

References

1. Handrick, V.; Vogt, T.; Frolov, A. Profiling of hydroxycinnamic acid amides in *Arabidopsis thaliana* pollen by tandem mass spectrometry. *Anal. Bioanal. Chem.*, **2010**, *398* (7–8), 2789–2801. https://doi.org/10.1007/s00216-010-4129-2.

2. Li, Z.; Zhao, C.; Zhao, X.; Xia, Y.; Sun, X.; Xie, W.; Ye, Y.; Lu, X.; Xu, G. Deep annotation of hydroxycinnamic acid amides in plants based on ultra-high-performance liquid chromatography-high-resolution mass spectrometry and its *in silico* database. *Anal. Chem.*, **2018**, *90* (24), 14321–14330. https://doi.org/10.1021/acs.analchem.8b03654.
