# Supplementary material for: Poison or Potion: Effects of Sunflower Phenolamides on Bumble Bees and Their Gut Parasite
Source: Biology (Basel). 2022 Apr 1;11(4):545. doi: 10.3390/biology11040545 (PMC9030180; doi:10.3390/biology11040545)
Supplement: Supplementary file 1 [file biology-11-00545-s001.zip › Protocol S2.pdf]

Protocol S2. Pollen diet preparation.

Phenolamide extract from sunflower pollen was obtained from ground sunflower pollen pellets by Soxhlet extraction using methanol at 100°C for 30 h. The methanolic extract was then filtrated and evaporated to dryness using a rotavapor (IKA RV8). The extract was finally dissolved in aqueous ethanol solution (1:1 v/v) before addition to the control willow pollen in proportions that mimic phenolamide concentrations of sunflower pollen diet. All treatment diets contained aqueous ethanol (1:1 v/v; 26 – 34 µL/diet g) to control for potential effects of the solvent (see Table A for diet formula). The total phenolamide content of willow pollen pellets, sunflower pollen pellets and phenolamide extract were analysed in triplicates by HPLC-MS/MS (triplicates of 20 – 40 mg) for quantification (expressed as triferuloyl spermidine equivalent, TSE). We found that willow pollen contained 23.21 ± 3.22 mg TSE/g, sunflower pollen 54.8 ± 3.74 mg TSE/g and phenolamide extract 161.22 ± 2.24 mg TSE/g (mean ± SD).

Table S4. Diet formula. In every treatment, the quantities shown here enabled to feed 15 microcolonies at the onset of the experiment (*i.e.*, when each microcolony was provided with 1 g of pollen candy).

	**Diet treatments**		
	Control diet (willow)	Natural diet (sunflower)	Supplemented diet (willow added with phenolamide extract)
**Pollen (g)**	15 (willow)	15 (sunflower)	15 (willow)
**65% sugar solution (numbers of drops)**	8	8	0
**Aqueous ethanol (v:v, 1:1) (mL)**	1.5	1	0
**Distilled water (mL)**	5.5	3	0
**Phenolamide extract (mL)**	0	0	7
**Final candy mass (g)**	22.23	19.29	23
**Ethanol in final candy (µL/g)**	34	26	29
**Pollen in final candy (g/g)**	0.67	0.78	0.65
**Phenolamides in final candy (mg/g)**	0	42.62	42.37
