# Supplementary material for: Poison or Potion: Effects of Sunflower Phenolamides on Bumble Bees and Their Gut Parasite
Source: Biology (Basel). 2022 Apr 1;11(4):545. doi: 10.3390/biology11040545 (PMC9030180; doi:10.3390/biology11040545)
Supplement: Supplementary file 1 [file biology-11-00545-s001.zip › Protocol S3.pdf]

Protocol S3. Implementation of parasite reservoirs.

Nine wild queens of *Bombus terrestris* were collected from the Mont Panisel (Mons, Belgium) on the 12th of March 2021 and placed in individual plastic boxes (10 × 16 × 16 cm) with willow pollen and 65% sugar solution (w/w) provided *ad libitum*. Their faeces were separately mounted on a microscope slide and screened using a light microscope (BA210, Motic; Hong Kong, China) at 400-fold magnification for the presence of the common parasites *Crithidia bombi* (Euglenozoa: Kinetoplastea: Trypanosomatidae), *Nosema bombi* (Microsporidia: Nosematidae), *Apicystis bombi* (Apicomplexa: Neogregarinida) and *Sphaerularia bombi* (Nematoda: Tylenchoidea: Allantonematidae). Six queens harboured the targeted parasite *C. bombi*. All the queens were free of *N. bombi* and *A. bombi* but three *Crithidia*-infected queens were also infected with the nematode *S. bombi* (eggs and third-stage juveniles observed in the faeces). Despite that *S. bombi* has never been observed in bumble bee workers, we decided to discard these queens prior to colony inoculation (*i.e.*, three *C. bombi*-infected queens left). Five commercial colonies were imported from Biobest *bvba* (Westerlo, Belgium) to be used as parasite reservoirs. Faeces from the three infected queens were collected on an every-two-day basis for 24 days, mixed with 65% sugar solution (w/w) and poured in bottle caps that were provided inside the colonies (*i.e.*, each colony was inoculated 12 times). The five colonies developed an infection but three were more severely infected and therefore used for further microcolony inoculations.
