# Supplementary material for: Poison or Potion: Effects of Sunflower Phenolamides on Bumble Bees and Their Gut Parasite
Source: Biology (Basel). 2022 Apr 1;11(4):545. doi: 10.3390/biology11040545 (PMC9030180; doi:10.3390/biology11040545)
Supplement: Supplementary file 1 [file biology-11-00545-s001.zip › Table S1.pdf]

Table S1. Model selection table showing candidate models for each analysis. Models in italic are the ones within the 95% confidence set that were used for model averaging.

**Pollen collection** ~ Diet * Parasite * Day + (1 | Colony:Microcolony) (Gamma distribution, log link)		**AICc**	**ΔAICc**	**Weight**
	Diet * Parasite * Day	1242.8	7.21	0.026
	Diet * Parasite	1509.6	273.98	0
	*Diet * Day*	1235.6	0	0.974
	Diet	1504.8	269.18	0
	Parasite * Day	1352.1	116.47	0
	Parasite	1564.6	328.95	0
	Day	1350.3	114.73	0
	Null	1562.6	326.99	0

**Syrup collection** ~ Diet * Parasite * Day + (1 | Colony:Microcolony) (Gamma distribution, log link)		**AICc**	**ΔAICc**	**Weight**
	*Diet * Parasite * Day*	3515.8	3.24	0.165
	Diet * Parasite	4582.9	1070.35	0
	*Diet * Day*	3512.6	0	0.835
	Diet	4577.8	1065.22	0
	Parasite * Day	3986.1	473.52	0
	Parasite	4609.7	1097.16	0

	Day	3985.5	472.99	0
	Null	4608.0	1095.43	0

**Larval ejection** ~ Diet * Parasite + (1 ǀ Colony:Microcolony) (Binomial distribution, logit link)		**AICc**	**ΔAICc**	**Weight**
	*Diet * Parasite*	632.2	0	0.777
	Diet	634.7	2.49	0.223
	Parasite	681.9	49.72	0
	Null	682.6	50.34	0

**Pollen efficacy** ~ Diet * Parasite + (1 ǀ Colony) (Gamma distribution, log link)		**AICc**	**ΔAICc**	**Weight**
	*Diet * Parasite*	-90.8	4.22	0.108
	Diet	-95.0	0	0.892
	Parasite	-7.7	87.3	0
	Null	-9.9	85.11	0

**Pollen dilution** ~ Diet * Parasite + (1 ǀ Colony) (Gamma distribution, log link)		**AICc**	**ΔAICc**	**Weight**
	*Diet * Parasite*	272.7	4.5	0.095
	Diet	268.2	0	0.905
	Parasite	318.0	49.8	0
	Null	316.5	48.24	0

**Worker mortality** ~ Diet * Parasite + (1│Colony) (Cox Proportional Hazard Model)		**AICc**	**ΔAICc**	**Weight**
	*Diet * Parasite*	415.2	0.82	0.194
	Diet	414.3	0	0.292
	Parasite	414.6	0.32	0.249
	Null	414.5	0.20	0.265

**Number of eggs** ~ Diet * Parasite + (1│Colony) (Negative binomial distribution, log link)		**AICc**	**ΔAICc**	**Weight**
	Diet * Parasite	644.0	5.48	0.026
	Diet	639.7	1.25	0.212
	Parasite	638.5	0	0.396
	Null	638.7	0.16	0.366

**Number of non-isolated larvae** ~ Diet * Parasite + (1│Colony) (Negative binomial distribution, log link)		**AICc**	**ΔAICc**	**Weight**
	*Diet * Parasite*	699.6	5.87	0.047
	Diet	693.8	0	0.886
	Parasite	701.2	7.44	0.021
	Null	699.7	5.93	0.046

**Number of pre-defecating larvae** ~ Diet * Parasite + (1 ǀ Colony:Microcolony) (Poisson distribution, log link)		**AICc**	**ΔAICc**	**Weight**
	*Diet * Parasite*	472.1	0	0.73
	Diet	474.1	1.99	0.27
	Parasite	522.1	49.93	0
	Null	520.0	47.85	0

**Number of post-defecating larvae** ~ Diet * Parasite + (1 ǀ Colony:Microcolony) (Poisson distribution, log link)		**AICc**	**ΔAICc**	**Weight**
	*Diet * Parasite*	307.8	5.21	0.069
	Diet	302.6	0	0.931
	Parasite	357.2	54.64	0
	Null	355.3	52.74	0

**Number of pupae** ~ Diet * Parasite + (1 ǀ Colony) (Negative binomial distribution, log link)		**AICc**	**ΔAICc**	**Weight**
	*Diet * Parasite*	452.9	0	0.645
	Diet	454.1	1.20	0.355
	Parasite	506.3	53.37	0
	Null	504.2	51.24	0

**Number of non-emerged males** ~ Diet * Parasite + (1ǀ Colony) (Poisson distribution, log link)		**AICc**	**ΔAICc**	**Weight**
	*Diet * Parasite*	185.1	3.79	0.084
	Diet	181.3	0	0.559
	Parasite	185.0	3.63	0.091
	Null	182.8	1.49	0.266

**Number of emerged males** ~ Diet * Parasite + (1ǀ Colony) (Negative binomial distribution, log link)		**AICc**	**ΔAICc**	**Weight**
	*Diet * Parasite*	574.4	5.71	0.054
	Diet	568.7	0	0.946
	Parasite	651.4	82.72	0
	Null	649.5	80.78	0

**Total mass of hatched alive offspring** ~ Diet * Parasite + (1ǀ Colony) (Gamma distribution, log link)		**AICc**	**ΔAICc**	**Weight**
	*Diet * Parasite*	398	5.01	0.076
	Diet	393.0	0	0.924
	Parasite	487.7	94.66	0
	Null	485.6	92.63	0

**Parasite load** ~ Diet * Day + (1 ǀ Colony:Microcolony) (Negative binomial distribution, log link)		**AICc**	**ΔAICc**	**Weight**
	*Diet * Day*	10459.4	0	0.991
	Diet	10747.0	287.61	0
	Day	10468.8	9.43	0.009
	Null	10752.3	292.91	0

**Fat body content** ~ Diet * Parasite * Caste + (1 ǀ Colony) (Gamma distribution, logit link)		**AICc**	**ΔAICc**	**Weight**
	*Diet * Parasite * Caste*	-966.2	0	0.994
	Diet * Parasite	-955.7	10.58	0.005
	Diet * Caste	-952.1	14.09	0.001
	Diet	-937.4	28.81	0
	Parasite * Caste	-916.0	50.20	0
	Parasite	-909.5	56.70	0
	Caste	-898.7	67.56	0
	Null	-890.5	75.75	0

**Centroid size** ~ Diet * Parasite + (1 ǀ Colony) (Gamma distribution, log link)		**AICc**	**ΔAICc**	**Weight**

	*Diet * Parasite*	449.1	0	0.998
	Diet	461.8	12.66	0.002
	Parasite	881.7	432.57	0
	Null	882.3	433.17	0

**Size FA** ~ Diet * Parasite + (1ǀ Colony) (Gamma distribution, log link)		**AICc**	**ΔAICc**	**Weight**
	*Diet * Parasite*	-4882.8	3.35	0.158
	Diet	-4886.2	0	0.842
	Parasite	-4841.5	44.73	0
	Null	-4842.4	43.76	0

**Size FA (without outliers)** ~ Diet * Parasite + (1ǀ Colony) (Gamma distribution, log link)		**AICc**	**ΔAICc**	**Weight**
	*Diet * Parasite*	-4849.3	4.30	0.068
	Diet	-4853.6	0	0.581
	Parasite	-4850.3	3.36	0.108
	Null	-4851.9	1.74	0.243

**Shape FA** ~ Diet * Parasite + (1ǀ Colony) (Gamma distribution, log link)		**AICc**	**ΔAICc**	**Weight**
	*Diet * Parasite*	-2331.6	5.59	0.058
	Diet	-2337.1	0	0.942

	Parasite	-2298.2	38.92	0
	Null	-2300.0	37.14	0

**Shape FA (without outliers)** ~ Diet * Parasite + (1∣ Colony) (Gamma distribution, log link)		**AICc**	**ΔAICc**	**Weight**
	Diet * Parasite	-2447.3	6.10	0.045
	Diet	-2453.4	0	0.955
	Parasite	-2421.9	31.45	0
	Null	-2423.9	29.49	0
