# Supplementary material for: Poison or Potion: Effects of Sunflower Phenolamides on Bumble Bees and Their Gut Parasite
Source: Biology (Basel). 2022 Apr 1;11(4):545. doi: 10.3390/biology11040545 (PMC9030180; doi:10.3390/biology11040545)
Supplement: Supplementary file 1 [file biology-11-00545-s001.zip › Table S2.pdf]

Table S2. Parameter outputs. Parameter estimates (PE), standard errors (SE) and 95% confidence intervals (CI) derived from model averaging across the confidence set of models. Significant parameters (*i.e.*, CI not crossing zero) are highlighted in italic.

**Pollen collection**	**Parameter / Level**	**PE**	**SE**	**Lower CI**	**Upper CI**
	Natural diet	-0.045	0.104	-0.249	0.158
	Supplemented diet	-0.407	0.102	-0.607	-0.206
	Day	0.073	0.006	0.061	0.084
	Natural diet:Day	-0.064	0.008	-0.080	-0.047
	Supplemented diet:Day	-0.004	0.008	-0.020	0.012
**Syrup collection**	**Parameter / Level**	**PE**	**SE**	**Lower CI**	**Upper CI**
	Natural diet	0.194	0.054	0.088	0.301
	Supplemented diet	0.079	0.054	-0.028	0.185
	Parasite	0.015	0.039	-0.062	0.092
	Day	0.056	0.002	0.051	0.061
	Natural diet:Parasite	-0.005	0.031	-0.066	0.055
	Supplemented diet:Parasite	-0.004	0.030	-0.063	0.055
	Day:Natural diet	-0.055	0.003	-0.061	-0.050
	Day:Supplemented diet	-0.018	0.003	-0.024	-0.012
	Day:Parasite	-0.001	0.004	-0.008	0.006
	Day:Natural diet:Parasite	0.001	0.003	-0.005	0.006
	Day:Supplemented diet:Parasite	0.001	0.004	-0.006	0.009
**Larval ejection**	**Parameter / Level**	**PE**	**SE**	**Lower CI**	**Upper CI**

	Natural diet	1.223	0.285	0.658	1.789
	Supplemented diet	-0.054	0.274	-0.598	0.490
	Parasite	-0.636	0.418	-1.459	0.188
	Natural diet:Parasite	0.575	0.455	-0.324	1.474
	Supplemented diet:Parasite	0.457	0.420	-0.374	1.287
**Pollen efficacy**	**Parameter / Level**	**PE**	**SE**	**Lower CI**	**Upper CI**
	Natural diet	-1.328	0.135	-1.596	-0.534
	Supplemented diet	0.060	0.121	-0.180	0.300
	Parasite	-0.011	0.060	-0.130	0.109
	Natural diet:Parasite	0.038	0.132	-0.222	0.298
	Supplemented diet:Parasite	0.012	0.081	-0.150	0.174
**Pollen dilution**	**Parameter / Level**	**PE**	**SE**	**Lower CI**	**Upper CI**
	Natural diet	0.343	0.050	0.244	0.441
	Supplemented diet	0.355	0.050	0.257	0.454
	Parasite	0.010	0.035	-0.060	0.079
	Natural diet:Parasite	-0.008	0.037	-0.082	0.066
	Supplemented diet:Parasite	-0.007	0.036	-0.079	0.064
**Worker mortality**	**Parameter / Level**	**PE**	**SE**	**Lower CI**	**Upper CI**
	Natural diet	0.486	0.771	-1.026	1.998
	Supplemented diet	0.663	0.954	-1.208	2.534
	Parasite	0.423	0.786	-1.117	1.962
	Natural diet:Parasite	-0.135	0.613	-1.335	1.066

	Supplemented diet:Parasite	-0.342	0.880	-2.067	1.384
**Number of eggs**	**Parameter / Level**	**PE**	**SE**	**Lower CI**	**Upper CI**
	Natural diet	-0.023	0.135	-0.292	0.246
	Supplemented diet	-0.110	0.250	-0.603	0.382
	Parasite	-0.150	0.237	-0.616	0.317
**Number of non-isolated larvae**	**Parameter / Level**	**PE**	**SE**	**Lower CI**	**Upper CI**
	Natural diet	0.292	0.164	-0.034	0.617
	Supplemented diet	-0.188	0.172	-0.530	0.154
	Parasite	-0.010	0.068	-0.145	0.124
	Natural diet:Parasite	0.011	0.081	-0.150	0.172
	Supplemented diet:Parasite	0.005	0.076	-0.146	0.155
**Number of pre-defecating larvae**	**Parameter / Level**	**PE**	**SE**	**Lower CI**	**Upper CI**
	Natural diet	-3.018	0.668	-4.338	-1.698
	Supplemented diet	-0.568	0.386	-1.333	0.197
	Parasite	-0.580	0.470	-1.507	0.346
	Natural diet:Parasite	1.380	1.022	-0.632	3.393
	Supplemented diet:Parasite	0.642	0.591	-0.526	1.810
**Number of post-defecating larvae**	**Parameter / Level**	**PE**	**SE**	**Lower CI**	**Upper CI**
	Natural diet	-2.744	0.467	-3.673	-1.815

	Supplemented diet	-0.104	0.243	-0.587	0.379
	Parasite	-0.023	0.118	-0.255	0.210
	Natural diet:Parasite	-0.023	0.265	-0.549	0.503
	Supplemented diet:Parasite	0.028	0.159	-0.285	0.342
**Number of pupae**	**Parameter / Level**	**PE**	**SE**	**Lower CI**	**Upper CI**
	Natural diet	-2.069	0.455	-2.968	-1.170
	Supplemented diet	-0.409	0.222	-0.849	0.030
	Parasite	-0.309	0.280	-0.861	0.243
	Natural diet:Parasite	0.767	0.691	-0.593	2.127
	Supplemented diet:Parasite	0.346	0.351	-0.347	1.039
**Number of non-emerged males**	**Parameter / Level**	**PE**	**SE**	**Lower CI**	**Upper CI**
	Natural diet	-0.566	0.573	-1.693	0.219
	Supplemented diet	-0.401	0.417	-1.224	0.422
	Parasite	-0.028	0.175	-0.375	0.319
	Natural diet:Parasite	0.111	0.432	-0.739	0.961
	Supplemented diet:Parasite	0.015	0.207	-0.396	0.427
**Number of emerged males**	**Parameter / Level**	**PE**	**SE**	**Lower CI**	**Upper CI**
	Natural diet	-1.699	0.191	-2.079	-1.318
	Supplemented diet	-0.363	0.119	-0.600	-0.125
	Parasite	-0.008	0.049	-0.106	0.089
	Natural diet:Parasite	0.005	0.089	-0.172	0.182

	Supplemented diet:Parasite	0.003	0.056	-0.108	0.114
**Total mass of hatched alive offspring**	**Parameter / Level**	**PE**	**SE**	**Lower CI**	**Upper CI**
	Natural diet	-1.918	0.155	-2.225	-1.611
	Supplemented diet	-0.410	0.146	-0.700	-0.120
	Parasite	-0.014	0.072	-0.155	0.128
	Natural diet:Parasite	0.029	0.127	-0.221	0.280
	Supplemented diet:Parasite	0.018	0.097	-0.175	0.210
**Parasite load**	**Parameter / Level**	**PE**	**SE**	**Lower CI**	**Upper CI**
	Natural diet	0.002	0.180	-0.350	0.355
	Supplemented diet	0.441	0.170	0.109	0.774
	Day	0.064	0.005	0.054	0.074
	Natural diet:Day	-0.010	0.008	-0.025	0.004
	Supplemented diet:Day	-0.011	0.007	-0.025	0.002
**Fat body content**	**Parameter / Level**	**PE**	**SE**	**Lower CI**	**Upper CI**
	Natural diet	-0.826	0.155	-1.130	-0.523
	Supplemented diet	-0.757	0.154	-1.060	-0.455
	Parasite	-0.466	0.157	-0.774	-0.157
	Caste	-0.459	0.158	-0.767	-0.150
	Natural diet:Parasite	0.335	0.212	-0.080	0.750
	Supplemented diet:Parasite	0.229	0.213	-0.189	0.646
	Natural diet:Caste	0.582	0.216	0.159	1.005

	Supplemented diet:Caste	0.259	0.212	-0.156	0.674
	Parasite:Caste	-0.012	0.217	-0.437	0.413
	Natural diet:Parasite:Caste	-0.032	0.300	-0.620	0.556
	Supplemented diet:Parasite:Caste	-0.027	0.292	-0.600	0.546
**Centroid size**	**Parameter / Level**	**PE**	**SE**	**Lower CI**	**Upper CI**
	Natural diet	-0.267	0.012	-0.290	-0.243
	Supplemented diet	-0.030	0.012	-0.053	-0.007
	Parasite	0.015	0.012	-0.008	0.038
	Natural diet:Parasite	0.032	0.017	0.000	0.065
	Supplemented diet:Parasite	0.004	0.017	-0.029	0.036
**Size FA**	**Parameter / Level**	**PE**	**SE**	**Lower CI**	**Upper CI**
	Natural diet	0.374	0.162	0.055	0.694
	Supplemented diet	0.990	0.170	0.655	1.324
	Parasite	-0.001	0.080	-0.159	0.157
	Natural diet:Parasite	-0.030	0.133	-0.292	0.232
	Supplemented diet:Parasite	0.048	0.159	-0.265	0.361
**Size FA (without outliers)**	**Parameter / Level**	**PE**	**SE**	**Lower CI**	**Upper CI**
	Natural diet	0.197	0.180	-0.157	0.552
	Supplemented diet	0.060	0.115	-0.168	0.287
	Parasite	-0.008	0.059	-0.125	0.109
	Natural diet:Parasite	-0.015	0.085	-0.182	0.151

	Supplemented diet:Parasite	0.006	0.069	-0.130	0.142
**Shape FA**	**Parameter / Level**	**PE**	**SE**	**Lower CI**	**Upper CI**
	Natural diet	0.182	0.043	0.098	0.266
	Supplemented diet	0.272	0.043	0.188	0.356
	Parasite	0.001	0.015	-0.028	0.030
	Natural diet:Parasite	-0.002	0.021	-0.044	0.040
	Supplemented diet:Parasite	0.001	0.021	-0.039	0.042
**Shape FA (without outliers)**	**Parameter / Level**	**PE**	**SE**	**Lower CI**	**Upper CI**
	Natural diet	0.181	0.031	0.120	0.241
	Supplemented diet	0.124	0.031	0.063	0.185
