# Supplementary material for: Poison or Potion: Effects of Sunflower Phenolamides on Bumble Bees and Their Gut Parasite
Source: Biology (Basel). 2022 Apr 1;11(4):545. doi: 10.3390/biology11040545 (PMC9030180; doi:10.3390/biology11040545)
Supplement: Supplementary file 1 [file biology-11-00545-s001.zip › Table S3.pdf]

Table S3. Characterisation of phenolamide profiles in sunflower nectar and pollen. No phenolamides were found in sunflower leaves and petals.

**Tissue**	**Compound**	**Molecular formula [M–H]-**	**Calculated mass [M–H]-**	**Δ ppm**
Nectar				
	N,N′-diferuloyl spermidine	$[C_{27}H_{34}N_3O_6]^-$	496,2448	0
	N,N′,N″-tricoumaroyl spermidine	$[C_{34}H_{36}N_3O_6]^-$	582,2606	0,3
	N,N′,N″-dicoumaroyl feruloyl spermidine	$[C_{35}H_{38}N_3O_7]^-$	612,2704	1
	N,N′,N″,N‴-tetracoumaroyl spermine	$[C_{46}H_{49}N_4O_8]^-$	785,3549	0,1
	N,N′,N″,N‴-tricoumaroyl feruloyl spermine	$[C_{47}H_{51}N_4O_9]^-$	815,3654	0,2
Pollen				
	N,N′,N″-tricoumaroyl spermidine	$[C_{34}H_{36}N_3O_6]^-$	582,2625	3,6
	N,N′,N″-dicoumaroyl feruloyl spermidine	$[C_{35}H_{38}N_3O_7]^-$	612,2734	3,9
	N,N′,N″,N‴-tetracoumaroyl spermine	$[C_{46}H_{49}N_4O_8]^-$	785,3571	2,7
	N,N′,N″,N‴-tricoumaroyl feruloyl spermine	$[C_{47}H_{51}N_4O_9]^-$	815,3744	10,8
